# Causal Forest Machine Learning Analysis of Parkinson’s Disease in Resting-State Functional Magnetic Resonance Imaging

**DOI:** 10.3390/tomography10060068

**Published:** 2024-06-06

**Authors:** Gabriel Solana-Lavalle, Michael D. Cusimano, Thomas Steeves, Roberto Rosas-Romero, Pascal N. Tyrrell

**Affiliations:** 1 Department of Computing, Electronics, and Mechatronics, Universidad de las Américas Puebla Santa Catarina Mártir, San Andrés Cholula, Puebla 78210, Mexico; gabriel.solanale@udlap.mx (G.S.-L.); roberto.rosas@udlap.mx (R.R.-R.); 2 Department of Medical Imaging, University of Toronto, Toronto, ON M5S 1A1, Canada; 3 Institute of Medical Science, University of Toronto, Toronto, ON M5S 1A1, Canada; michael.cusimano@unityhealth.to; 4 Division of Neurosurgery, Unity Health Toronto, St. Michael’s Hospital, Toronto, ON M5B 1W8, Canada; 5 Division of Neurology, Unity Health Toronto, St. Michael’s Hospital, Toronto, ON M5B 1W8, Canada; thomas.steeves@unityhealth.to; 6 Department of Statistical Sciences, University of Toronto, Toronto, ON M5S 1A1, Canada

**Keywords:** computer-assisted, Causal Forest, functional Magnetic Resonance Imaging, Machine Learning, Multiple Correspondence Analysis, Parkinson’s disease detection

## Abstract

In recent years, Artificial Intelligence has been used to assist healthcare professionals in detecting and diagnosing neurodegenerative diseases. In this study, we propose a methodology to analyze functional Magnetic Resonance Imaging signals and perform classification between Parkinson’s disease patients and healthy participants using Machine Learning algorithms. In addition, the proposed approach provides insights into the brain regions affected by the disease. The functional Magnetic Resonance Imaging from the PPMI and 1000-FCP datasets were pre-processed to extract time series from 200 brain regions per participant, resulting in 11,600 features. Causal Forest and Wrapper Feature Subset Selection algorithms were used for dimensionality reduction, resulting in a subset of features based on their heterogeneity and association with the disease. We utilized Logistic Regression and XGBoost algorithms to perform PD detection, achieving 97.6% accuracy, 97.5% F_1_ score, 97.9% precision, and 97.7%recall by analyzing sets with fewer than 300 features in a population including men and women. Finally, Multiple Correspondence Analysis was employed to visualize the relationships between brain regions and each group (women with Parkinson, female controls, men with Parkinson, male controls). Associations between the Unified Parkinson’s Disease Rating Scale questionnaire results and affected brain regions in different groups were also obtained to show another use case of the methodology. This work proposes a methodology to (1) classify patients and controls with Machine Learning and Causal Forest algorithm and (2) visualize associations between brain regions and groups, providing high-accuracy classification and enhanced interpretability of the correlation between specific brain regions and the disease across different groups.

## 1. Introduction

Parkinson’s disease (PD) is a synucleinopathy that affects various regions of the brain as it progresses [1]. It is characterized by dopamine depletion and alterations in different neural structures and neurochemical systems of the brain [2]. Functional Magnetic Resonance Imaging (fMRI) is a non-invasive neuroimaging technique that medical experts have used to assess the functional state of brain regions implicated in the motor and non-motor symptoms of PD [3]. This imaging modality has been useful to (1) detect functional abnormalities across the whole brain using connectivity and graph analysis, (2) distinguish between PD patients and healthy controls, and (3) track the progression of the disease in longitudinal studies [4].

In recent years, Artificial Intelligence (AI) has assisted healthcare professionals (HP) in the detection of neurological diseases through the early detection pathological areas [5]. To analyze the fluctuations in blood flow in the brain, shown on resting-state functional MRI (rs-fMRI), machine learning (ML) algorithms have proven to be valuable tools to (1) discover spatial patterns, (2) find dynamic functional connectivity patterns, and (3) develop diagnostics or forecasting tools [6].

One of the challenges when using ML with medical images is the high number of features available to train these algorithms. Reducing the number of features, also known as feature selection or dimensionality reduction, offers models of lower complexity and easier to interpret. Dimensionality reduction applied to medical image analysis can lead to a better understanding of the relationships between features and the Regions of Interest (ROIs) that contribute the most to the decision-making process during ailment detection. Approaches such as Radiomics are based on extracting many descriptors and then selecting the most useful ones to train the ML algorithms [7]. During rs-fMRI analysis, some of the most used techniques to reduce dimensionality and enhance ML performance include linear dimensionality reduction like PCA [8], non-linear dimensionality reduction like Laplacian eigenmaps [9], and ML-based dimensionality reduction like Support Vector Machines [10,11], LASSO [12,13,14], and tree-based algorithms [15].

In recent years, comparisons between correlation-based and causal-based feature selection have been of interest, as the latter techniques present different advantages over traditional correlation-based feature selection. Some advantages of causal-based methods include their ability to select the most relevant features that have a meaningful impact on the outcome, improve the generalization of the model with unseen data, and provide more consistent results, sometimes at the expense of accuracy and computational complexity [16].

In this work, we propose a methodology that leverages causal-based and correlation-based feature selection along with ML to detect PD in fMRI signals. First, we use a recent technique named Causal Forest (CF) [17], which has been used for estimating causal effects in multiple areas including medicine [18], education [19], finance [20], and software simulations [21] among others. CF is an efficient algorithm capable to rank features based on the frequency with which features are used to split data across trees. The goal is to reduce the dimensionality of the dataset and select only the features that most affect the outcome (PD or healthy). Once we have a smaller subset of features, we use the traditional correlation-based Wrapper Feature Subset Selection (WFSS) algorithm, which is a resource-intensive algorithm, to identify the best subset for a specific classifier.

To assist the interpretability of the results, we leverage statistical tools to visualize and interpret the results and provide an analysis of the Regions of Interest (ROIs) in the brain that have a strong association with the disease, according to a data-based analysis of the extracted features. Therefore, expert analysis can focus on these regions.

The objectives of this work included providing a set of features to describe the activations in the brain of PD patients, thereby achieving (1) high accuracy and (2) interpretability of PD detection with ML, and (3) the ability to visualize the ROIs and scores associated with the disease according to the Unified Parkinson’s Disease Rating Scale.

## 2. Materials and Methods

**Figure 1:**
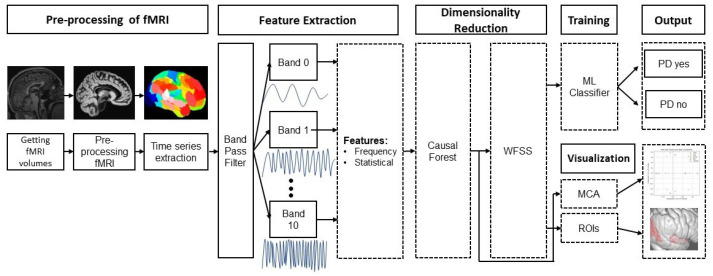
Methodology for PD detection and subsequent interpreting analysis of PD with rs-fMRI.

Figure 1 shows an overview of the proposed approach, which consists of eight stages: (1) image pre-processing, (2) generation of a 200-time series that describes the temporal variations of the average gray level content in each region, (3) a bank of band-pass filters that separate each time series into 10 bands, (4) feature extraction from each filtered time series, (5) selection of the most relevant features with CF, (6) selection of the features that achieve the best classification performance with the Wrapper Feature Subset Selection (WFSS) algorithm, (7) classification to identify an individual as PD positive or negative, and (8) Multiple Correspondence Analysis (MCA) to associate each group with those brain regions with the highest average causal effect. This methodology was implemented in Python version 3.10.9. The code for this publication is publicly available at the GitHub page: https://github.com/GabrielSolana29/fmri_causalForest (accessed on 2 June 2024).

### 2.1. Data Set

The rs-fMRI images from PD patients and controls were obtained from the Parkinson’s Progression Markers Initiative (PPMI) data set [22]. Initially, 217 PPMI fMRI images were selected for this study, coming from 46 male and 22 female patients. The Unified Parkinson’s Disease Rating Scale (UPDRS) test, available on the PPMI site, was used to rate the severity and progression in 68 PD patients. Since the PPMI data set does not contain enough fMRI images from controls for a balanced study, different public data sets were used to obtain additional fMRI images. The additional data sets were obtained from the 1000 Functional Connectomes project (FCP), which contains rs-fMRI images from 33 sites [23]. Both datasets are publicly available. Some patients contributed various images to the data set since they participated in different stages of the disease. Table 1 shows the distribution of images in different groups. Each data set was categorized by sex, age range, and repetition time (TR). The voxel size ranged from 3 to 4 mm, and the number of fMRI frames ranged from 175 to 265, which accounts for 6.3 to 8.8 minutes of scanning time. Data from each of the individual datasets from the 1000 FCP that were used in this work can be found in Table S1 of the Supplementary Material.

To handle variations in data quality, resolution, and acquisition methods, crucial in machine learning applications, four data processing strategies were incorporated to obtain a reliable and robust method. (1) *Standardization* is a preprocessing technique that was used to make the data more uniform by centering data around the mean and scaling data to obtain a unit variance. (2) *Image registration* applies transformations like rotation, scaling and flipping to align images taken with different sensors. (3) Instead of using raw image voxel values, *feature extraction* involves the generation of relevant features from images under different domains (time, frequency), which can be more robust to image variations. (4) An *ensemble of feature selection methods* was used to reduce the impact of image variations and improve performance and robustness.

### 2.2. Image Pre-Processing

The fMRI volumes from the PPMI dataset were transformed to the BIDS format to standardize them. The tools used to pre-process images were fMRI-prep version 21.0.2 and Nipype version 1.6.1 [24], which create a robust and reproducible pre-processing pipeline that uses different packages such as FSL, FreeSurfer, and AFNI. The pipeline can perform co-registration, normalization, warping, noise component extraction, segmentation, and skull stripping with state-of-the-art techniques [25]. The output of the fMRI-prep pipeline includes the anatomical and the BOLD files.

The first step in the pipeline was to pre-process and skull-strip anatomical T1w structural MRI. Then, the tissues were segmented, and spatial normalization was performed. Finally, the T1w images underwent surface reconstruction to accurately map functional activations from fMRI onto the participant’s brain surface [25]. BOLD images underwent reference image estimation to create a brain mask for the BOLD signals, head motion estimation, and slice time correction, where the slices were re-aligned in time to the middle of each repetition time. Susceptibility distortion correction was performed to account for the differences in the field inside the scanners. The surface created with the T1w image was used to calculate the alignment with the Echo Planar Image (EPI), then it was mapped to the standard spaces of the T1w mask, and the surfaces are generated based on the template space [25].

### 2.3. Generation of Time Series and Feature Extraction

For each individual, the cerebral cortex was divided into 200 regions according to the partition in the atlas generated by Schaefer et al. [26]. A time series was extracted from each brain region, which carried the temporal variation of the voxel gray intensity within the brain region. The total number of features extracted was 11,600 features per participant. These features were categorized into three groups: voxel gray intensity, frequency and connectivity.

The voxel gray intensity carried within the time series extracted from a brain region was analyzed to calculate six features that represent the brightness levels of voxels in an image, making them easy to interpret: (1) *mean value*, (2) *variance*, (3) *skewness*, (4) *kurtosis*, (5) *average auto-correlation*, and (6) *maximum wave height* of the time series, which is the vertical distance between the crest and the trough. The total number of *voxel gray intensity features* was 200 brain time series per participant × six features per time series = 1200 features per participant.

The Fourier Transform of each time series was computed to generate its frequency spectrum and extract frequency features, which provide information about the spatial distribution of voxel gray intensities at different frequency scales indicating differences in brain tissue density. The spectrum of each time series was analyzed within a bandwidth ranging from 0.01 to 0.15 Hz, where there is a significant presence of neural fluctuations and an absence of higher frequencies that contain noise from respiration and cardiac signals [27]. The frequency spectrum of a brain time series was divided into ten bands, each of 0.014 Hz. From each band, four frequency features were calculated: (1) band energy, (2) band dominant frequency, (3) variance, (4) average auto-correlation. In addition to the extraction of four features from each spectrum band, two global features were calculated by considering the entire frequency range (from 0.01 to 0.15 Hz) that includes the ten bands: (1) *dominant frequency*, (2) *energy percentage of dominant frequency*. Thus, the total number of frequency features was *200 signal spectra per participant* × (*ten bands per spectrum* × *four features per band *+ *two features per entire spectrum*) = *8400 features per participant*.

At each brain region, a connectivity analysis was conducted to calculate the average correlation between each band and a baseline sinusoidal signal with a frequency corresponding to the central frequency of the band under analysis. The maximum overlap between the baseline sinusoidal and each band’s time series was determined. The total number of connectivity features was *2000 connectivity features* per participant.

### 2.4. Feature Selection with Causal Forest

Feature selection was used to obtain a subset from the original set of 11,600 features. Feature selection reduces computational complexity, improves classification performance and enhances interpretability by finding the most relevant features while discarding noisy, redundant, or irrelevant features [17,28,29]. Classification performance is improved by removing irrelevant features that introduce noise and decrease accuracy. In addition, fewer features make it easier to understand and interpret the relationships between inputs and outputs. In this research Causal Forests (CF) and Wrappers Feature Subset Selection (WFSS) were combined to provide a stable and reliable feature subset.

Causal forests (CF) use the same principles for Random Forests (RF) [30,31], which are based on various nested binary decisions implemented with if-then-else statements that recursively split data into smaller subsets based on the values of features. The hierarchical structure of a single binary decision-making process consists of nodes and branches and that is why it is named decision tree. RFs consist of an ensemble of decision trees, where each tree is trained independently on a subset of the training data and a random subset of features [32]. RFs aggregate the decisions taken by various decision trees to make a final one, reduce overfitting, and improve generalization performance if compared to a single decision tree.

Causal forests (CF) train each tree on a random subset of features at each split in the same manner as RF. This randomization allows growing diverse trees and reduces the chance of overfitting to specific features. CFs use splitting criteria based on causal inference rather than entropy, which is used as criterion in RF. CF splitting criteria assess the relationship between features and outcomes. Thus, CFs grow more effective trees and focus on relevant features for estimating causal inference. After determining the relevance of each feature, these are ranked based on their contribution to the average contribution to Causal Forest. CFs were implemented using the open-source software developed by Microsoft Research [33].

CFs handle data heterogeneity that refers to the presence of a diversity of features within the data. CFs effectively capture heterogeneity in features across different groups, identifying specific features and allowing an understanding of how features vary across different classes.

After feature set reduction with CF, the number of features still remained high, so further reduction was conducted by using WFSS [34], an algorithm that searches for the optimal feature subset to achieve optimum PD detection. WFSS starts with an empty set of selected features. It then generates candidate feature subsets using a heuristic search method. Each candidate feature subset is used to train a classifier. The classification performance of the classifier is evaluated using a performance metric to select the subset that achieves the best performance.

### 2.5. Classification

The participant’s features were fed to a classifier to classify an fMRI as corresponding to a PD patient or a control individual. In this research, PD detection was implemented using two classification models: the logistic classifier [35] and extreme gradient boosting (XGBoost) [36]. These two classification models have been used in various computer-assisted diagnosing applications [37,38,39].

The logistic classifier is a classification model that is built using a conditional probability function $P\left(y=1|x,\omega \right)=\frac{1}{1+e^{-\omega^{T}\;x}}$ and its complement P(y=1|x,ω)=1−P(y=0|x,ω), where x is the feature vector extracted from one participant, y=1 corresponds to the outcome “PD positive”, y=0 corresponds to the outcome “PD negative”, and ω is a vector that contains the model parameters. To train the logistic classifier, a loss function was defined and maximized using the maximum likelihood algorithm.

Extreme Gradient Boosting (XGBoost) builds an ensemble of decision trees by starting with weak trees. Each decision tree is trained sequentially to correct the errors from previous trees using a gradient boosting strategy so that the new tree is stronger than the previous ones. Each new tree is trained by minimizing the residuals from previous trees, where the residuals are the difference between the predicted values and the desired ones. XGBoost optimizes the parameters of decision trees using gradient descent optimization techniques. Once individual decision trees are trained, their classification outcomes are aggregated using a weighted voting scheme to obtain the final outcome.

### 2.6. PD Detection Performance Assessment

PD detection performance was evaluated with *k*-fold cross-validation [40]. The data set was divided into *k* subsets or folds so that the classifier is trained and tested *k* times, an important strategy when the dataset is relatively small. Each time, a different fold is used for evaluation and the remaining folds are used for training. In *k*-fold cross-validation, all the feature vectors are used for training and testing, which leads to a more reliable evaluation of the classification performance. The performance metrics used to assess classification performance were accuracy, recall, $F_{1}$ score, and precision. These metrics were averaged across *k* iterations to obtain a more reliable estimate of the classification performance. In addition, *k*-fold cross-validation can be used to select the optimal set of model parameters while providing a reliable estimate of the classification performance.

Nested cross-validation uses a double loop: an outer loop to assess the quality of the model, and an inner loop for model selection. The outer loop is repeated *ℓ* times to generate *ℓ* different outer test sets and *ℓ* different outer test sets. At each iteration of the outer loop, the outer training set is further divided into *k* folds. Thus, the total number of trained models is *ℓ* × *k*. At each iteration of the outer loop, only the best inner model is evaluated on the outer test set. The total number of performance estimates is *ℓ*, and these are are averaged to assess the quality of the classification model.

Since some patients contributed with various images at different stages of the disease, using the same patient for training and testing was avoided. All the studies coming from one patient were in the same fold so that the information of that patient was only used for training or for testing at each of the *k* iterations.

### 2.7. Multiple Correspondence Analysis

Multiple Correspondence Analysis (MCA) is a statistical technique used to uncover associations and dependencies between characteristics or variables and groups or populations [41,42,43]. Medical images are characterized by features of high dimension and MCA helps in reducing the dimensionality of these variables while preserving relevant information and allowing visualization and interpretation. Visualization in a lower dimensional space enables the exploration of relationships between variables and populations simultaneously. This leads to insights into brain regions associated with PD patients.

Since features may have different scales and levels of variability, these data is normalized, ensuring that each variable contributes equally to the analysis. For each brain region, MCA computes the frequency or number of occurrences of each particular characteristic. MCA then performs a transformation on the data to retain only the two most significant dimensions based on their inertia values, which captures the maximum amount of variance. MCA calculates the (x,y) coordinates in the reduced-dimensional space. These coordinates represent the positions of brain regions and groups (male PD patients, male controls, female PD patients, female controls) along the MCA dimensions so that data can be visualized in the reduced-dimensional space using points to represent populations and brain regions and their positions reflect their similarities. MCA can assist in categorizing images into distinct groups, which corresponds to PD patients and controls, men and women.

## 3. Results

### 3.1. Parkinson Disease Detection Performance Assessment

**Figure 2:**
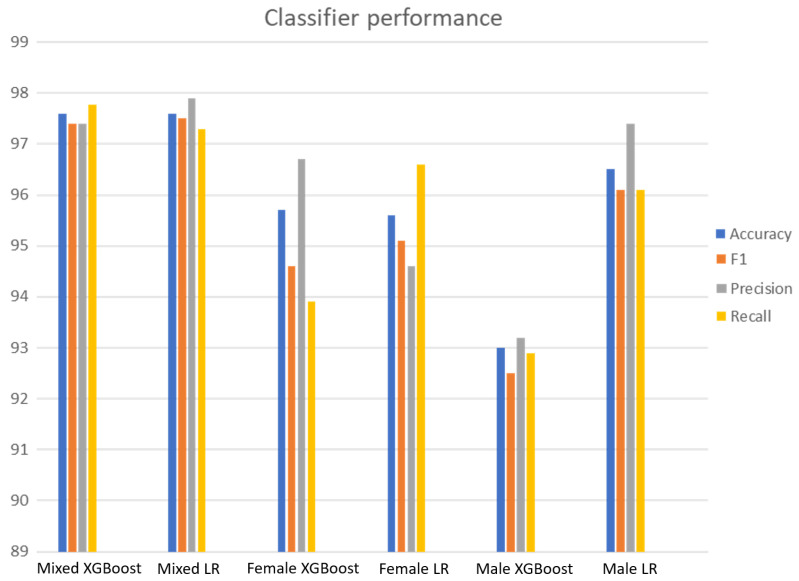
PD detection performance in three populations: female, male, and mixed. For each population, PD detection was implemented using two classifiers, XGBoost and logistic regression (LR), using nested cross-validation. Four metrics were used to measure detection performance: accuracy, F1 score, precision and recall.

Figure 2 shows the PD detection performance in six experiments conducted using two classifiers (XGBoost and logistic regression) in three populations: women, men, and mixed. For each set of experiments, four metrics were used to assess PD detection performance: accuracy, F1 score, precision, and recall. These results are shown in Figure 2. The complete set of experiments using nested cross-validation for each group can be found in Tables S2–S7 of the Supplementary Material.

### 3.2. Identification of the Most Relevant Characteristics for PD Detection

Feature extraction provided a set of 11,600 features per participant. However, the use of all the features provided classifiers with little discriminative power for PD detection. The selection of the most relevant features helped to improve classification performance and reduce model complexity. The number of features was reduced with CF, which kept the most relevant features in terms of impact across two groups, PD patients and controls. Once CF reduction was conducted, the feature set was further reduced for each classifier by running WFSS. Finally, PD detection performance was evaluated and the results are shown in Figure 2. CF and WFSS considerably reduced the feature set as shown in Table 2 that specifies (1) reduction percentage of the feature set, (2) number of features, and (3) PD detection accuracy for three population (female, male, mixed) using two classifiers (logistic regression, XGBoost).

A 98% reduction means that only 2% of the top-ranked features by CF were chosen. CF was run five times during the PD detection evaluation, setting the feature set reduction percentage to 99.9%, 99.5%, 99%, 98%, and 96%. Table 2 shows the reduction percentage and the number of features corresponding to the highest PD detection performances in six experiments. The most predominant group of features for PD detection corresponded to voxel gray intensity, followed by frequency features. Once features were selected with CF and WFSS, 56% (based on the XGBoost classifier) and 60.4% (LR) of the selected features corresponded to gray intensity, 39.2% (XGBoost) and 36.3% (LR) corresponded to frequency, 4.8% (XGBoost) and 3.3% (LR) corresponded to connectivity.

### 3.3. Brain Regions with the Highest Average Contribution to Causal Forest in Four Populations

To interpret fMRI data from each participant, 11,600 features were extracted from 200 brain regions that were localized according to the partition in the atlas generated by Schaefer et al. [26]. Thus, 58 features were extracted from each brain region. Causal forest assigned a score to each feature and ranked it according to its contribution during the split of decision trees. Since causal forest build binary trees in a random manner, causal forest was run ten times to select those features that were highly ranked at least during four runs. Once CF ranked all the features, the top 4% were selected. Table 3 shows the percentage of selected features by CF from each group of features (gray intensity, frequency and connectivity) and for each population (female contro, female PD patient, male control, male PD patient).

Since features were extracted from 200 brain regions, each brain region is characterized by a specific set of highly ranked features. In each brain region, the average of the scores assigned by CF to the top-ranked features was calculated. These average rankings were used to determine the regions with the highest average contribution to CF. The complete list of ROIs selected by CF, their ranking, and frequency of appearance in each group are shown in Figure S1 of the Supplementary Material.

**Figure 3:**
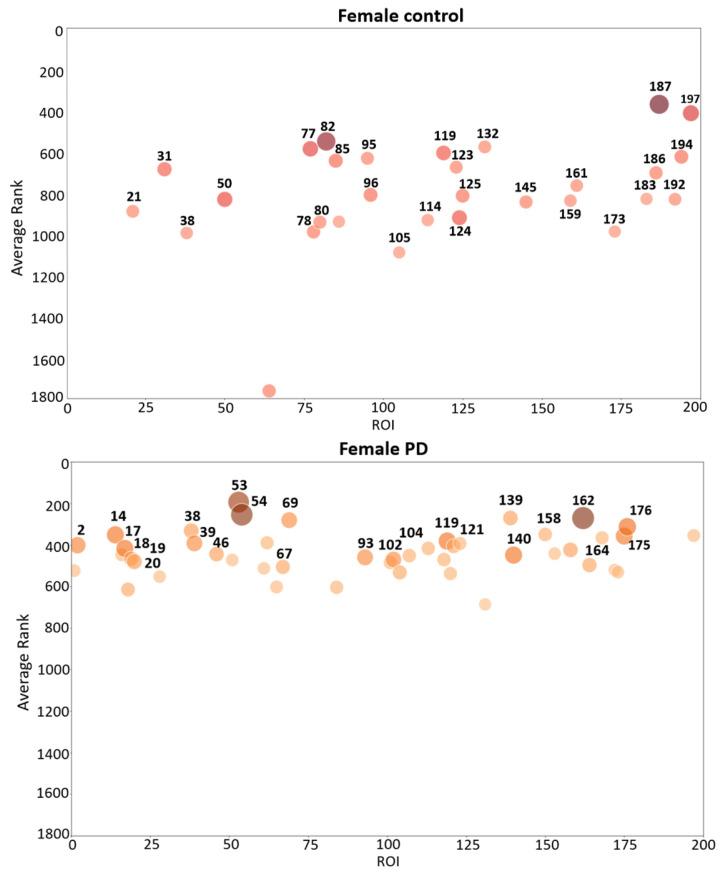
Bubble plot that shows brain region indexes (*X*-axis), region average rankings (*Y*-axis), and number brain features (bubble size and color intensity) for female PD patients (lower panel) and female controls (upper panel). The parcellation of the ROIs shown in the figure come from the Schaefer 2018 atlas [26].

**Figure 4:**
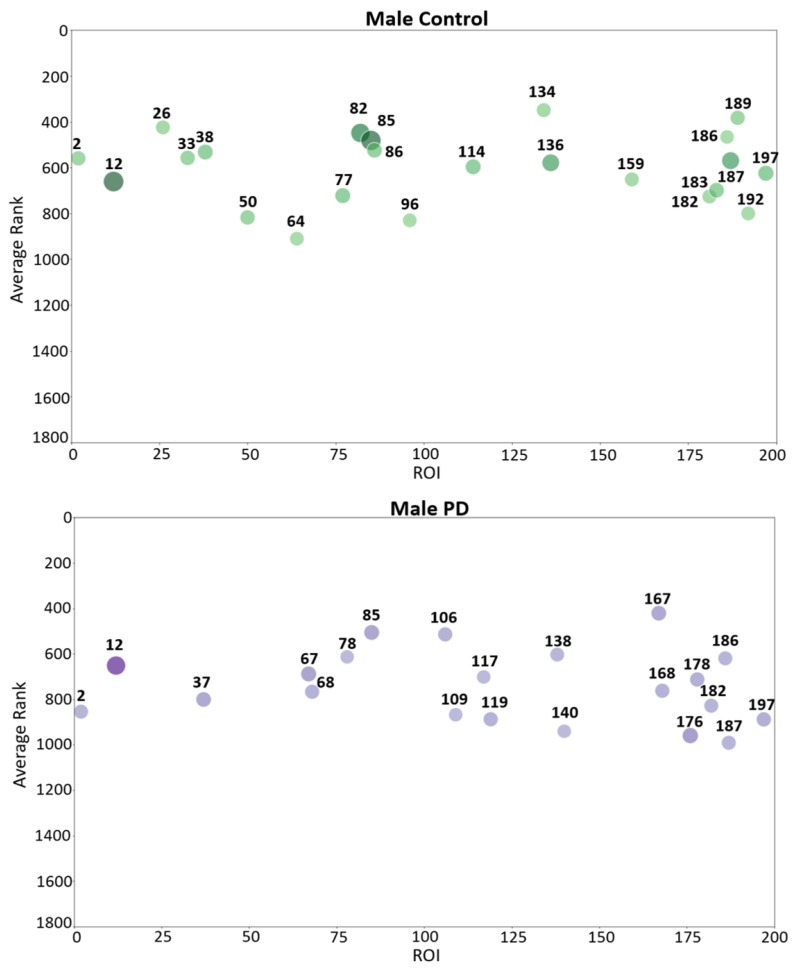
Bubble plot that shows brain region indexes (*X*-axis), region average rankings (*Y*-axis), and number of region features (bubble size and color intensity), for male PD patients (lower panel) and male controls (upper panel). The parcellation of the regions shown in this figure comes from the Schaefer 2018 atlas [26].

Bubble plots are a useful tool to visually identify brain regions with the highest average contribution to causal forest in each group (female PD patients, female controls, male PD patients, and male controls). The bubble plots in Figure 3 assist in visualizing those brain regions from which highly ranked features come from. These plots show the average ranking (*y* axis) and the number of relevant features (bubble size) in various brain regions for two groups: female PD patients (left panel) and female controls (right panel). For each brain region, a bubble plot shows the relationship between (1) the index of the brain region, represented by the *x* axis; (2) its corresponding average ranking, represented by the *y* axis; and (3) the number of top-ranked features, represented by the dot size and color intensity. The greater the *y* coordinate the higher the region average ranking. The larger the bubble size the larger the number of top-ranked features in the brain region. Each brain region was assigned an integer index (*x* axis) that takes values from 1 to 200 since the cerebral cortex was divided into 200 regions. Figure 4 shows the corresponding bubble plots for PD male patients (left panel) and male controls (right panel). The brain regions shown in the bubble plots correspond to the top-ranked regions which were later used to conduct multiple correspondence analysis.

### 3.4. Association between Brain Regions and Populations

**Figure 5:**
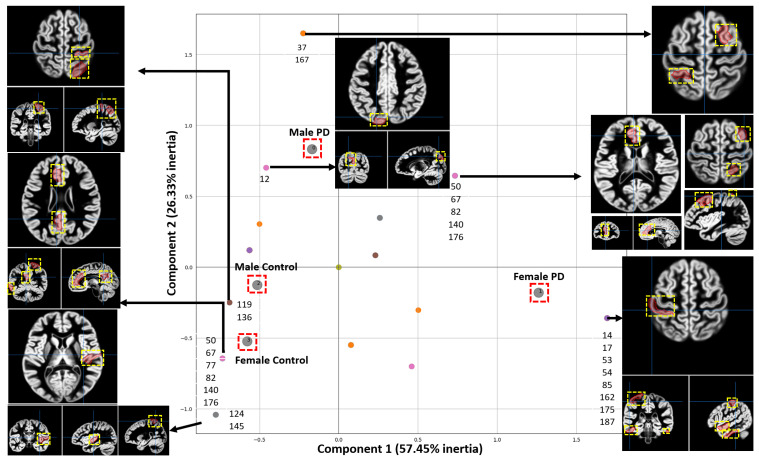
Associations between brain regions and groups. Each point represents an association between a group and the regions. The two most important Principal Dimensions are shown, which account for 83.78% of the total inertia. Each axis accounts for a different amount of inertia, where the weight of the *X*-axis is higher than the one of the *Y*-axis. The parcellation of the regions shown in this figure comes from the Schaefer 2018 atlas [26]. The ROIs are shown as red areas inside the yellow dashed box.

Causal forests provided an understanding of the relationship among features, brain regions and each specific group (female PD patients, female controls, male PD patients, male controls). MCA was then used to identify and visually interpret associations between each group and brain regions (200 brain regions were localized according to the atlas generated by Schaefer et al. [26]) by projecting these variables as points on a two-dimensional space, as shown in Figure 5. This information is valuable to understanding the conditions under which a specific brain region is more informative about a group. The bubble plots of Figure 3 and Figure 4 show associations between brain regions and each group separately; however, interdependencies among groups are not shown. On the other hand, MCA can handle all the groups simultaneously in a single graph.

Each point enclosed in a red square in the MCA plot of Figure 5 represents a specific group. The position of each point in the MCA plot is determined by the relationships among groups and brain regions with the highest average contribution to causal forest. Points closer to each other indicate that these points are associated with similar brain regions. Since the *x* axis contains more inertia, the higher the separation from the center on this axis, the more relevant the associations. In some points, a list of the associated brain regions is shown. For some brain regions their corresponding fMRI views are shown. The usefulness of MCA is based upon closeness between points from the same set in a low-dimensional space since proximity between points means that observations associated with these points are themselves similar.

**Figure 6:**
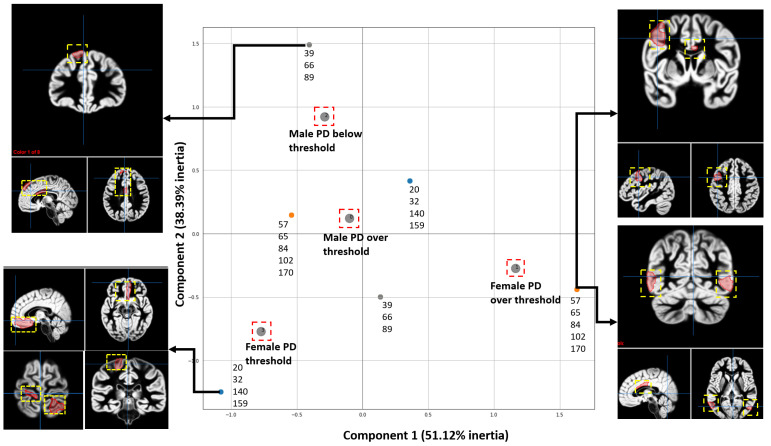
MCA analysis shows the associations between the scores of the motor section of the UPDRS test and the most relevant ROI resulting from running CF with the data from the patients with PD from the PPMI. Four groups were studied, including Males and Females with positive results from the third section UPDRS and Males and Females with negative results from the motor test. The most relevant regions are shown in the brain illustrations. The two Principal Dimensions with the highest inertia are shown, which account for 89.51% of the total inertia. Since each axis accounts for different amount of inertia, the weight of the *X*-axis is bigger than the one of the *Y*-axis. The ROIs are shown as red areas inside the yellow dashed box.

MCA was also used to visualize the associations between brain regions and groups (female PD patients, female controls, male PD patients, PD controls) by considering patients with a total score above 23.7 (threshold) in the third section of the UPDRS scale, as shown in Figure 6. This scale was used to correlate regions for each group based on the rankings given by causal forest. The criterion for obtaining the threshold was based on the work of [44], which specifies that the second stage of the Hoehn and Yahr scale (HYS) is met approximately at that score.

The following steps were done to find associations between the fMRI regions and the UPDRS scores using CF and MCA: (1) Features were extracted from the fMRI of each patient. (2) Data was labelled into one of four classes or groups, including female PD patients with scores below and above the threshold and male PD patients with scores below and above the threshold. (3) The CF algorithm was run to obtain the scores for each feature. (4) MCA was performed to visualize the results.

## 4. Discussion

This paper presents a new methodology to (1) assist PD detection by analyzing fMR images and (2) find relationships between brain regions and each of four groups: female PD patients, female controls, male PD patients, male controls. The fMRI studies for these groups were obtained from the PPMI and FCP public datasets. Although this research was focused on PD, the same methodology can be applied to assist the diagnosis of other neurodegenerative disorders by analyzing fMRI data.

### 4.1. Data Set

The majority of control images come from data sets different to the one with images from PD patients since capturing fMRI scans from controls with the same acquisition system can be very difficult due to lack of interest in participating in such studies, time or cost. In such cases, the use of different control data sets that are representative can be a practical alternative. Pre-processing was applied to images from different data sets, mainly standardization and image registration. Data pre-processing helps a classification model not to detect differences in data quality, resolution, and acquisition methods as differences between PD patients and controls. The incorporation of control data acquired from diverse settings strengthens the statistical validity of the analysis.

### 4.2. Selection of the Most Relevant Causal Characteristics in Each Group for PD Detection

The first step towards a reduction of the feature set and an increase in PD detection performance consisted of ranking and selecting features according to their contribution to causal forest during the split of decision trees. This ranking process mainly depends on feature relevance, which corresponds to feature heterogeneity. Once the feature set is reduced to keep a small percentage of the top ranked features by CF, WFSS was used to select the best feature subset for each classifier, resulting in dimensionality reduction and an increased PD classification performance. The reason for conducting CF followed by wrappers to reduce the feature set is that WFSS is much more time consuming than CF, where the latter is faster and requires a smaller amount of memory.

From the three families of features utilized in this work (voxel gray intensity, frequency-based, and connectivity-based features), gray intensity features were the most predominant according to CF. The WFSS algorithm mostly tends to choose frequency features.

### 4.3. PD Detection

Two ML models (LR and XGBoost) were used to implement PD detection, which reached the highest accuracy when using feature subsets that contained at most 4% of the original feature set, as shown in Table 2. The highest PD detection accuracy (97.6%) was reached in a mixed population. The lowest accuracy was reached when detecting PD in men (93%).

Table 4 shows the performance of various methods, including the proposed one. The Table describes these methodologies in terms of dataset, framework, and the corresponding results. The PPMI dataset is the most used one by the scientific community and it is mostly used to predict PD progression.

None of the other methods conducted experiments on male and female populations. The size of the PPMI dataset (70 female and 147 male patients) allowed for experiments to be conducted on separate populations, women and men. However, a limitation is the reduced number of fMRI studies coming from controls (4 female and 18 male controls). The use of data from the 1000 FCP allowed the generation of two balanced datasets: The data for women consisted of (1) PPMI studies from 22 patients and 4 controls and (2) 1000 FCP studies from 18 controls. The data for men consisted of (1) PPMI studies from 46 patients and 18 controls and (2) 1000 FCP studies from 28 controls.

PD detection performance was higher in women than in men. This result coincides with those obtained with MCA since the correspondence analysis indicates that the main characteristics of the female population have a discriminative power (PD patient vs. control) greater than that of the characteristics extracted from men.

### 4.4. Associations between Brain Regions and Groups of Participants

To interpret fMRI data from 200 brain regions, MCA projects the relationships between brain regions and each of four groups (female PD patients, female controls, male PD patients, male controls) into a two-dimensional space containing the two most important principal dimensions. MCA highlights for each group those brain regions with the highest average contribution to Causal Forest. The associations along the *x* axis are the most relevant due to the higher inertia. MCA shows the brain regions associated with each group according to CF. Figure 5 shows that some of the regions, relevant and indicative in male PD patients, include the left hemisphere visual peripheral extrastriate superior cortex, the right hemisphere lateral dorsal prefrontal cortex, and different regions from the left hemisphere temporal parietal. In female PD patients, the most relevant and indicative regions are located in the right second quadrant of the MCA plot as shown in Figure 5 and these regions include the right and left hemisphere limbic temporal pole, the right hemisphere lateral dorsal prefrontal cortex, and multiple regions from the right and left hemisphere somatomotor region.

Figure 6 shows the associations between brain regions and each group of participants according to the UPDRS scores. These results show that the proposed approach can identify the relationships between features extracted from fMRI studies and the scores of the progression of the disease’s motor symptoms. Figure 6 illustrates the ROIs most associated with one group (female above/below threshold or male below/above threshold) according to the CF ranking when compared to the other three groups. We are visualizing only the regions that are characteristic of one group; for example, the group of females above threshold shows that the right central visual cortex was selected as a ROI. This does not mean that other regions, such as the left hemisphere’s somatomotor network in the group of females below the threshold, are unaffected.

### 4.5. Limitations

A valid concern exists where nested cross-validation does not increase confidence in the PD detection results and introduces additional parameters that may be over-tuned to the training data without proper held-out validation on unseen data. This may lead to limited ability to reveal generalizing patterns about the disease state. The controls that provided data for this work are substantially younger than the patients, and AI models are well-known to be sensitive to age-related changes in MR imaging. Distinguishing between younger and older patients could account for a substantial portion of the contrast between PD patients and controls. For these reasons, this is a proof of concept that remains to be validated.

### 4.6. Parkinsonism vs. Parkinson as Future Work

The proposed method could be used to discriminate between patients with PD and patients with Parkinsonism, which is a more complex problem. As more studies from PD patients become available, applying the proposed method to a longitudinal study with multiple images from each patient could shed some light on the progression of brain affectations from the disease.

Understanding subtypes of PD (tremor-dominant and akinetic-rigid) can be enhanced with machine learning techniques to provide insights into the neural mechanisms underlying the different manifestations of PD [50]. Causal forest along with multiple correspondence analysis might facilitate the differentiation between akinetic-rigid and tremor-dominant subtypes, which differ in symptomatology and progression by selecting and identifying features in imaging data for identification of PD subtypes.

## 5. Conclusions

This work proposes a method for (1) classifying fMRI studies as PD positives or negatives and (2) visualizing the relationships between brain regions and four groups in a two-dimensional plot. The four groups include fMRI from women with and without PD, and men with and without PD. The characteristics extracted from the fMRI included voxel gray intensity, frequency-based, and connectivity features extracted from 200 regions. CF was used for dimensionality reduction by selecting those features that contribute the most to the representation of each particular group (male PD patients, male controls, female PD patients, female controls). The results showed that different brain regions are associated with a specific population. In addition, the results showed that regions that contributed the most in PD patients with a high UPDRS motor score are different from those that corresponded to patients with low scores. The classification of fMRI studies achieved a performance above 92% in four metrics (accuracy, $F_{1}$ score and recall) by analyzing a reduced number of features and maintaining a high level of interpretability.

## Figures and Tables

**Table 1:** Distribution of images used for PD detection and analysis. The images are grouped in terms of data sets, number of patients, sex, age range, and repetition time.

Data Set	PD		Control			
	**Female**	**Male**	**Female**	**Male**	**Age**	**TR**
PPMI	70	147	4	18	38–77	2.4
1000 Functional Connectome Project	0	0	103	101	19–65	2–2.3
Total	70	147	107	119	19–77	2–2.4

**Table 2:** Feature set reductions that correspond to the best PD detection accuracy using nested cross-validation. The original number of features per patient was 11,600. After CF reduction, WFSS was used to tailor the subset to each classification model.

Dataset	CF Reduction	Number of Features	PD Detection Accuracy
Mixed XGBoost	98%	192	0.976
Mixed LR	96%	26	0.976
Female XGBoost	99%	445	0.957
Female LR	96%	55	0.956
Male XGBoost	96%	16	0.930
Male LR	96%	37	0.965

**Table 3:** Percentage of features selected by CF from each group of features (voxel gray intensity, frequency, and connectivity) for each population (female control, male control, female PD patient, male PD patient).

Family of Features	CF Female Control	CF Male Control	CF Female PD	CF Male PD
Voxel gray intensity	59.21%	31.41%	43.05%	31.5%
Frequency	35.43%	65.52%	56.53%	64.82%
Connectivity	5.36%	3.07%	0.41%	3.67%

**Table 4:** A comparison with other PD detection methods.

Author and Year	Dataset	Framework	Performance
Kazeminejad et al., 2017 [45]	Resting state fMRI data from 18 healthy controls and 19 patients	Global graph theoretical metrics were extracted and used as features to a support vector machine classifier	Accuracy of 95% with leave-one-out cross-validation
Guo et al., 2022 [46]	84 subjects (56 in stage 2 and 28 in stage 1) from PPMI data set	Long short-term memory (LSTM) network to characterize early stages of PD	Accuracy of 71.63% with 10-fold stratified cross-validation
Nguyen et al., 2020 [47]	PPMI data from 82 PD subjects are used to predict clinical severity and progression at 1 year, 2 years, and 4 years	High- and low-progression is classified with Gradient Boosting, ElasticNet and SVM	Positive predictive values up to 71% and negative predictive values up to 84%
Cao et al., 2020 [12]	Private data from fifty healthy controls and 70 PD patients	6664 features are extracted and fed to SVM	Accuracy 100%
Rubbert et al., 2019 [48]	Private data from 42 PD patients and 47 controls	Boosted Logistic Regression models were trained with correlation matrices	Accuracy 76.2%, sensitivity 81%, specificity 72.7%
Fariah Haq et al., 2020 [49]	University of British Columbia data from fifteen control participants and seventeen PD patients	Graph theoretic features were extracted and fed to an SVM classifier	Sensitivity 94%
Proposed method	PPMI data from 46 male and 22 female patients. PPMI data from 18 male and 4 controls. Data from the 1000 Functional Connectcomes project dataset: 28 male and 18 female controls	Logistic Classifier and XGBoost, gray intensity and frequency features, feature selection	Accuracy 95.7% (women), 96.5% (men) and 97.6% (mixed). Precision 96.7% (women), 97.4% (men) and 97.4% (mixed). Recall 96.6% (women), 96.1% (men) and 97.7% (mixed). $F_{1}$ score 95.1% (women), 96.1% (men), 97.5% (mixed)

## Data Availability

This analysis used data openly available from PPMI. Data from Parkinson’s disease patients used in the preparation of this article were obtained from the Parkinson’s Progression Markers Initiative (PPMI) database (accessed on 25 June 2022) (www.ppmi-info.org/access-dataspecimens/download-data), RRID:SCR 006431. For up-to-date information on the study, visit www.ppmi-info.org; Data for healthy controls were obtained from the 1000 Functional Connectomes Project (accessed on 3 July 2022) (http://fcon_1000.projects.nitrc.org).

## Supplementary Materials

The following supporting information can be downloaded at: https://www.mdpi.com/article/10.3390/tomography10060068/s1.

## Abbreviations

The following abbreviations are used in this manuscript:

AI	Artificial Intelligence
CF	Causal Forest
EPI	Echo Planar Image
FCP	1000 Functional Connectomes Project
fMRI	Functional Magnetic Resonance Imaging
HP	Healthcare Professionals
HYS	Hoehn and Yahr scale
LASSO	Least Absolute Shrinkage and Selection Operator
MCA	Multiple Correspondence Analysis
ML	Machine Learning
PD	Parkinson’s Disease
PPMI	Parkinson’s Progression Markers Initiative
RF	Random Forest
ROI	Region of Interest
rs-fMRI	Resting-State Functional Magnetic Resonance Imaging
TR	Repetition Time
UPDRS	Unified Parkinson’s Disease Rating Scale
WFSS	Wrapper Feature Subset Selection
XGBoost	Extreme Gradient Boosting
