# Supplementary material for: Causal Forest Machine Learning Analysis of Parkinson’s Disease in Resting-State Functional Magnetic Resonance Imaging
Source: Tomography. 2024 Jun 6;10(6):894–911. doi: 10.3390/tomography10060068 (PMC11209036; doi:10.3390/tomography10060068)
Supplement: Supplementary file 1 [file tomography-10-00068-s001.zip › tomography-2953964-supplementary.pdf]

Supplementary Materials: Causal Forest Machine Learning Analysis of Parkinson's Disease in Resting-state Functional Magnetic Resonance Imaging.

Article: Causal Forest Machine Learning Analysis of Parkinson's Disease in Resting-state Functional Magnetic Resonance Imaging.

Corresponding author: Pascal N. Tyrrell

Email: pascal.tyrrell@utoronto.ca

Affiliation: University of Toronto

Journal: Tomography

Feature selection and Regions of Interest Association:

Figure S1 shows the Regions of Interest (ROI) with the highest average causal effect obtained by running the Causal Forest (CF) algorithm 10 times and averaging the results. For each population (Male PD, Female PD, Male control, and Female control) we present the ROI in the first column, the frequency of appearance (how many features selected by CF belong to the ROI) in the second column, the Average Rank (the average of the ranking in the CF selection out of 11,600 features, where higher is better) and the name of the ROI according to the atlas from Schaefer 2018. We only show the ROI that got selected with an average of at least 4 features highly ranked by CF.

	Top regions of interest															
	Male PD				Female PD				Male control				Female control			
Ranking	ROI	Frequency	Avg Rank	Name of the ROI	ROI	Frequency	Avg Rank	Name of the ROI	ROI	Frequency	Avg Rank	Name of the ROI	ROI	Frequency	Avg Rank	Name of the ROI
1	12	7.2	651.1	LH_VisPeri_ExStrSup_6	162	12.3	270.1	RH_Limbic_TempPole_2	12	8.1	660.6	LH_VisPeri_ExStrSup_6	187	9.4	370	RH_DefaultA_PFCm_1
2	176	5.1	959.2	RH_ContB_PFCld_2	54	11.9	253.9	LH_Limbic_TempPole_2	85	7.9	481	LH_DefaultB_Temp_3	82	8.7	551.2	LH_DefaultA_PFCm_3
3	67	4.9	687.91	LH_ContB_Temp_1	53	11.1	193.38	LH_Limbic_TempPole_1	82	7.1	448.4	LH_DefaultA_PFCm_3	197	6.7	414.1	RH_TempPar_1
4	85	4.8	505.3	LH_DefaultB_Temp_3	176	7.9	312.1	RH_ContB_PFCld_2	187	6.1	569.4	RH_DefaultA_PFCm_1	77	6.3	587.2	LH_DefaultA_PCC_1
5	167	4.7	420.5	RH_ContA_PFCd_1	119	7.7	380.8	RH_SomMotA_7	136	6	578.7	RH_DorsAttnA_SPL_4	50	6.1	838.9	LH_SalVentAttnB_PFCmp_1
6	37	4.7	800.3	LH_DorsAttnB_PostC_3	14	7.6	350.7	LH_SomMotA_2	197	4.9	624.2	RH_TempPar_1	124	5.9	922.1	RH_SomMotB_S2_1
7	178	4.5	712	RH_ContB_PFClv_2	17	7.6	416.5	LH_SomMotA_5	38	4.8	531.8	LH_DorsAttnB_PostC_4	119	5.7	607.2	RH_SomMotA_7
8	197	4.5	887.9	RH_TempPar_1	140	7.6	448.5	RH_DorsAttnB_PostC_4	114	4.8	596	RH_SomMotA_2	31	5.4	686.3	LH_DorsAttnA_TempOcc_3
9	106	4.4	513.7	RH_VisCent_ExStr_6	175	7.4	357	RH_ContB_PFCld_1	77	4.8	772.1	LH_DefaultA_PCC_1	85	5.2	645.1	LH_DefaultB_Temp_3
10	168	4.4	762	RH_ContA_PFCl_1	2	6.9	400.87	LH_VisCent_ExStr_2	86	4.7	523.8	LH_DefaultB_Temp_4	194	5.1	625.7	RH_DefaultC_IPL_1
11	68	4.4	766.4		39	6.6	391.5	LH_DorsAttnB_FEF_1	183	4.7	698.9	RH_ContC_Cingp_1	96	5.1	811.25	LH_DefaultC_IPL_1
12	119	4.4	887.3	RH_SomMotA_7	93	6.6	459.1	LH_DefaultB_PFCv_2	33	4.5	557.2	LH_DorsAttnA_SPL_2	125	5	815.62	RH_SomMotB_S2_2
13	186	4.3	619.2	RH_DefaultA_PCC_1	69	6.5	280.1	LH_ContB_PFCl_1	50	4.5	817.3	LH_SalVentAttnB_PFCmp_1	186	4.7	704	RH_DefaultA_PCC_1
14	182	4.3	827.8	RH_ContC_pCun_2	102	6.4	469.7	RH_VisCent_ExStr_2	189	4.4	382.4	RH_DefaultA_PFCm_3	78	4.7	991.1	LH_DefaultA_PCC_2
15	2	4.3	854	LH_VisCent_ExStr_2	20	5.9	479.79	LH_SomMotA_8	2	4.4	559.3	LH_VisCent_ExStr_2	64	4.7	1764.3	LH_ContA_PFCl_3
16	187	4.3	990.7	RH_DefaultA_PFCm_1	38	5.8	331.4	LH_DorsAttnB_PostC_4	134	4.2	348.7	RH_DorsAttnA_SPL_2	145	4.6	845.7	RH_SalVentAttnA_Ins_2
17	138	4.2	602.7	RH_DorsAttnB_PostC_2	139	5.6	269.9	RH_DorsAttnB_PostC_3	26	4.2	424.1	LH_SomMotB_Aud_6	95	4.5	633	LH_DefaultB_PFCv_4
18	78	4	612.6	LH_DefaultA_PCC_2	158	5.6	423.3	RH_Limbic_OFC_2	159	4.2	651.3	RH_Limbic_OFC_3	123	4.5	676.5	RH_SomMotA_11
19	117	4	701.1	RH_SomMotA_5	46	5.6	443.6	LH_SalVentAttnA_FrMed_2	181	4.2	725.8	RH_ContC_pCun_1	80	4.5	943.8	LH_DefaultA_PFCm_1
20	109	4	867.6	RH_VisPeri_ExStrSup_3	19	5.4	465.6	LH_SomMotA_7	192	4.1	800	RH_DefaultB_PFCd_1	132	4.4	577.8	RH_DorsAttnA_TempOcc_2
21	140	4	940.2	RH_DorsAttnB_PostC_4	164	5.4	496	RH_Limbic_TempPole_4	96	4.1	830	LH_DefaultC_IPL_1	21	4.4	891.1	LH_SomMotB_Aud_1
22					67	5.3	505.3	LH_ContB_Temp_1	64	4.1	910.1	LH_ContA_PFCl_3	161	4.3	765.9	RH_Limbic_TempPole_1
23					121	5.2	392.5	RH_SomMotA_9	186	4	465.9	RH_DefaultA_PCC_1	192	4.3	832.5	RH_DefaultB_PFCd_1
24					104	5.2	531.9	RH_VisCent_ExStr_4					159	4.2	839.28	RH_Limbic_OFC_3
25					18	5.1	614	LH_SomMotA_6					38	4.2	996.4	LH_DorsAttnB_PostC_4
26													114	4.1	933.6	RH_SomMotA_2
27													183	4	830.7	RH_ContC_Cingp_1
28													173	4	989.4	RH_ContB_IPL_1
29													105	4	1090.9	RH_VisCent_ExStr_5

Figure S1: Table showing the ranking of the ROI that contained at least 4 features highly ranked by the CF for each population. The table displays the frequency of appearance of ROI in the selected features, the average rank of the features, and the name of the ROI according to the Schaefer 2018 atlas are shown. To account for the stochastic nature of the CF, 10 iterations were conducted to obtain the results. Color code for the table is as follows: dark orange indicates that the feature is present in three of the four groups, light orange indicates the ROI was selected in two of the four groups, green color indicates the highest average ranked ROI for the group.

Information on control datasets:

The 1000 Functional Connectome Project contains more than 1,200 publicly available resting-state functional Magnetic Resonance Images (rs-fMRI) from 33 sites. Of these, images from the eight sites listed in Table S1 are used for the control participants in this work. These images were chosen because their characteristics, such as age, time points, and repetition time, most closely resemble those of the Parkinson's Progression Marker Initiative data.

Table S1: Data from the Control participants in this work. All datasets come from the 1000 Functional Connectome Project and are named as reported in the repository.

Dataset from the 1000 FCP	Female	Male	Age	TR
**Atlanta**	15	13	22-57	2.02
**Milwaukee-b**	31	12	46-65	2.18
**Leipzig**	21	16	20-42	2.3
**Queensland**	8	11	20-34	2.1
**Palo Alto**	15	2	20-46	2
**Berling-Margulies**	13	13	23-44	2.3
**Bangor**	0	22	19-38	2
**Leiden 2180**	0	12	20-27	2.18
**Total**	103	101	19-65	2-2.3

Classification Results:

Tables S2 and S3 show the PD detection performance in populations of female and male individuals using XGBoost and LR, respectively. The first column of each Table specifies the reduction percentage of the entire feature set with CF. The second column shows the number of selected features with WFSS. The last four columns present the values of the four performance metrics that were used: accuracy, F1 score, precision, and recall. The best results are highlighted in boldface.

Table S2: PD detection performance in a population combining both female and male individuals was assessed using an XGBoost classifier. The entire feature set was reduced with CF to obtain five feature set reduction percentages, shown in the first column. The final number of selected features with WFSS is shown in the second column.

CF reduction	WFSS features	Accuracy	F1	Precision	Recall
99.9	5	0.810	0.805	0.810	0.817
99.5	43	0.938	0.934	0.933	0.939
99	67	0.964	0.963	0.962	0.965
98	192	0.976	0.974	0.974	0.977
96	449	0.961	0.961	0.961	0.963

Table S3: PD detection performance in a population combining both female and male individuals was assessed using a LR classifier. CF was applied to obtain five reduction percentages (first column). WFSS was used to select a reduced number of features (second column).

CF reduction	WFSS features	Accuracy	F1	Precision	Recall
99.9	8	0.878	0.874	0.877	0.876
99.5	25	0.899	0.896	0.907	0.900
99	105	0.931	0.930	0.935	0.932
98	26	0.976	0.975	0.979	0.973

96	443	0.881	0.878	0.879	0.882

Tables S4 and S5 show the PD detection performance in a female population with XGBoost and LR, respectively. Tables S6 and S7 present the PD detection performance in a male population with XGBoost and LR, respectively. The first column of each Table specifies the feature set reduction with CF. The second column shows the reduced number of features with WFSS. The performance metric values are shown in the last four columns. The best results are highlighted in boldface.

Table S4: PD detection performance in a female population using an XGBoost classifier. CF was applied to obtain five reduction percentages (first column). WFSS was used to select a reduced number of features (second column).

**CF reduction**	**WFSS features**	**Accuracy**	**F1**	**Precision**	**Recall**
99.9	6	0.869	0.841	0.841	0.858
99.5	19	0.898	0.886	0.899	0.904
99	107	0.931	0.925	0.933	0.934
98	74	0.935	0.927	0.938	0.932
96	445	0.957	0.946	0.967	0.939

Table S5: PD detection performance in a female population using a LR classifier. CF was applied to obtain five reduction percentages (first column). WFSS was used to select a reduced number of features (second column).

**CF reduction**	**WFSS features**	**Accuracy**	**F1**	**Precision**	**Recall**
99.9	8	0.854	0.837	0.832	0.873
99.5	7	0.877	0.861	0.874	0.901
99	47	0.956	0.949	0.949	0.956
98	55	0.956	0.951	0.946	0.966
96	453	0.897	0.854	0.853	0.865

Table S6: PD detection performance in a male population using an XGBoost classifier. CF was applied to obtain five reduction percentages (first column). WFSS was used to select a reduced number of features (second column).

**CF reduction**	**WFSS features**	**Accuracy**	**F1**	**Precision**	**Recall**
99.9	5	0.821	0.809	0.822	0.818
99.5	6	0.895	0.889	0.894	0.893
99	4	0.905	0.898	0.914	0.904
98	16	0.930	0.925	0.932	0.929
96	382	0.915	0.910	0.917	0.913

Table S7: PD detection performance in a male population using a LR classifier. CF was applied to obtain five reduction percentages (first column). WFSS was used to select a reduced number of features (second column).

**CF reduction**	**WFSS features**	**Accuracy**	**F1**	**Precision**	**Recall**
99.9	16	0.910	0.897	0.910	0.901
99.5	25	0.906	0.891	0.908	0.891
99	93	0.876	0.860	0.866	0.864
98	37	0.965	0.961	0.974	0.961
96	419	0.950	0.944	0.952	0.943
